# Study of the Oxygen Evolution Reaction at Strontium Palladium Perovskite Electrocatalyst in Acidic Medium

**DOI:** 10.3390/ijms21113785

**Published:** 2020-05-27

**Authors:** Areej A. Eskandrani, Shimaa M. Ali, Hibah M. Al-Otaibi

**Affiliations:** 1 Department of Chemistry, Faculty of Science, Taibah University, Madinah 3002, Saudi; a.eskandrani@gmail.com (A.A.E.); black.cat1900@hotmail.com (H.M.A.-O.); 2 Department of Chemistry, Faculty of Science, Cairo University, Giza 12613, Egypt

**Keywords:** perovskite, Sr_2_PdO_3_, catalyst, OER, electrochemical techniques

## Abstract

The catalytic activity of Sr_2_PdO_3_, prepared through the sol-gel citrate-combustion method for the oxygen evolution reaction (OER) in a 0.1 M HClO_4_ solution, was investigated. The electrocatalytic activity of Sr_2_PdO_3_ toward OER was assessed via the anodic potentiodynamic polarization and electrochemical impedance spectroscopy (EIS). The glassy carbon modified Sr_2_PdO_3_ (GC/Sr_2_PdO_3_) electrode exhibited a higher electrocatalytic activity, by about 50 times, in comparison to the unmodified electrode. The order of the reaction was close to unity, which indicates that the adsorption of the hydroxyl groups is a fast step. The calculated activation energy was 21.6 kJ.mol^−1^, which can be considered a low value in evaluation with those of the reported OER electrocatalysts. The Sr_2_PdO_3_ perovskite portrayed a high catalyst stability without any probability of catalyst poisoning. These results encourage the use of Sr_2_PdO_3_ as a candidate electrocatalyst for water splitting reactions.

## 1. Introduction

Electrochemical water splitting is the process of the decomposition of water into its components, hydrogen and oxygen, by applying an electric current in the presence of an electrolyte. It provides a green alternative route for producing hydrogen fuel, which can be utilized instead of non-renewable and environmentally harmful fossil fuels [1,2,3,4]. The oxygen evolution reaction (OER) and the anodic reaction of the water splitting process are the major challenges facing researchers. The kinetics of the OER involve complicated multi-electron steps with the prospective existence of several accompanying processes such as the dissolution of the metal oxide catalyst. Thus, the multitude and complexity of the OER mechanism can create difficulty in comprehending it clearly [5,6]. In addition, the OER requires the application of high overpotentials [7,8,9], leading to a decrease in the efficiency of the hydrogen production by the water splitting. The most common catalysts for the OER are IrO_2_/RuO_2_, Pt, and HfN which can be efficiently employed but with low stability and high cost [10,11]. Nowadays, researchers seek multi-functional catalysts, which are abundant, cost-effective, stable, and of high catalytic activity. Perovskites, mixed metal oxides possessing the general formula ABO_3_ where A is a lanthanide and B is a transition metal, have been extensively used as effectual catalysts for OER in alkaline [12,13,14,15,16,17,18,19] and acidic media [20,21,22,23,24]. Lu and co-workers have reported that the LaCoO_3_ perovskite demonstrated high electrocatalytic behavior due to its unique electronic structure, which facilitates the presence of the oxygen vacancies in a high concentration [25]. Oxygen vacancies can highly increase the intrinsic activity of the perovskite active sites for the electro-oxidation reactions [26,27,28,29,30,31].

The SrPdO_3_ perovskite, prepared for the first time by the citrate combustion methodology by our group, presented interesting electrocatalytic and sensing applications [32]. Furthermore, SrPdO_3_ has been used as an efficient catalyst for the hydrogen evolution reaction [32], which was subsequently implemented for the first time by our group as an electrochemical sensor for several neurotransmitters and hydrazine detection [33,34,35]. It was discovered that the nano-dispersed Pd sites within the stable perovskite matrix are more effectual than the electro-deposited Pd. Recently, the JCPDS card of the SrPdO_3_ perovskite (00-025-0908) has been replaced by the JCPDS card of Sr_2_PdO_3_ (00-028-1249). It was reported that the partial substitution at the A-site of Sr_2_PdO_3_ and the formation of the nano-composites of the Sr_2_PdO_3_/carbon nanotubes and Sr_2_PdO_3_/gold nanoparticles can further enhance its electro-sensing ability for glucose and some drugs [36,37,38]. The study of the electronic structure of SrPdO_3_ disclosed that it can exhibit a spin transition from low to high at a certain temperature, which is similar to the isoelectronic LaCoO_3_ [39], and is one of the main reasons for the enhanced electrocatalytic activity of LaCoO_3_ for the OER.

In this work, Sr_2_PdO_3_, prepared by the sol-gel citrate-nitrate combustion procedure, was employed as an electrocatalyst for the OER in an acidic medium. The electrocatalytic activity was examined by the potentiodynamic polarization (PP) and electrochemical impedance spectroscopy (EIS). The kinetic and thermodynamic evaluations were performed to identify the reaction order and the activation energy, respectively. The catalyst stability was also investigated to ensure a satisfactory performance.

## 2. Results and Discussion

### 2.1. Structural and Surface Characterizations of Sr_2_PdO_3_

**Figure 1:**
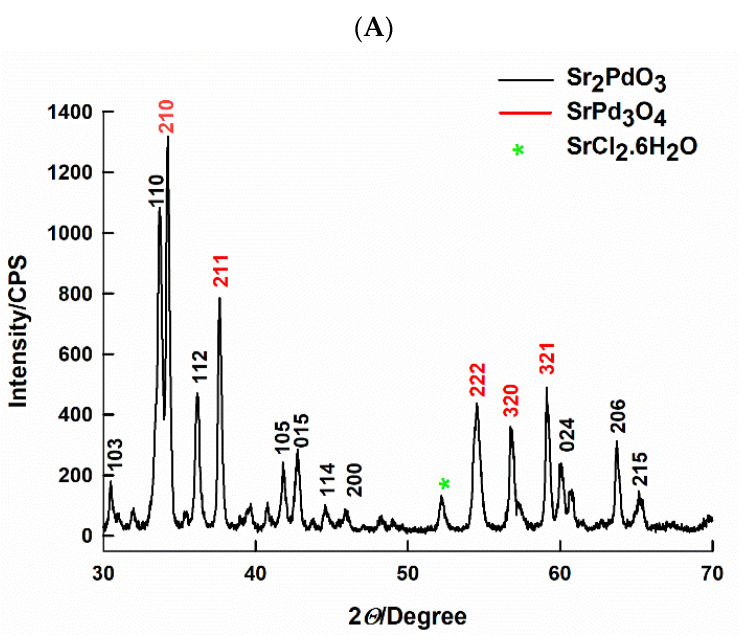

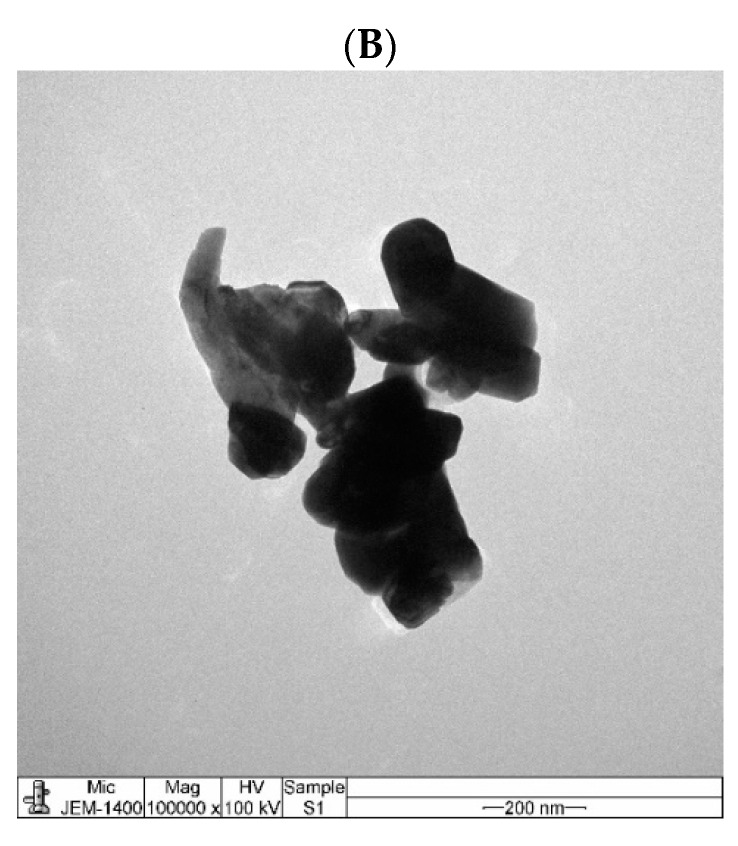
The (**A**) XRD pattern and (**B**) TEM image of Sr_2_PdO_3_ prepared by the citrate-method, miller indices (*h, l, k*) are showed.

Figure 1A illustrates the XRD spectrum of the strontium palladium perovskite, prepared by the citrate-combustion method at a pH value of 2 and at a calcination temperature of 750 °C for 3 h. The formation of the orthorhombic Sr_2_PdO_3_ perovskite is confirmed by the appearance of its theoretical major peak (110), JCPDS card number: 00-028-1249. However, the major phase of the fashioned sample is SrPd_3_O_4_, which is verified via the XRD pattern, displays a major peak at (210), and agrees with the theoretical value, Figure 1A. The formation of SrPd_3_O_4_, as a secondary phase, during the synthesis of Sr_2_PdO_3_, is well reported, due to the self-regeneration property of the Pd-based perovskites [40]. However, in the prepared sample, SrPd_3_O_4_ is the major phase; this is possibly because of the use of Pd^2+^ rather than the Pd^4+^ salt as a precursor during the synthesis. The calculated average particle size, according to the Scherrer equation [41], is 24.2 nm. The value of the measured BET surface area is 5.0 m^2^ g^−1^.

The morphology of the prepared Sr_2_PdO_3_ is observed by the TEM, Figure 1B. It is witnessed that the prepared perovskites consist of agglomerations of orthorhombic particles.

### 2.2. Examination of the Electrocatalytic Activity of Sr_2_PdO_3_ for OER

**Figure 2:**
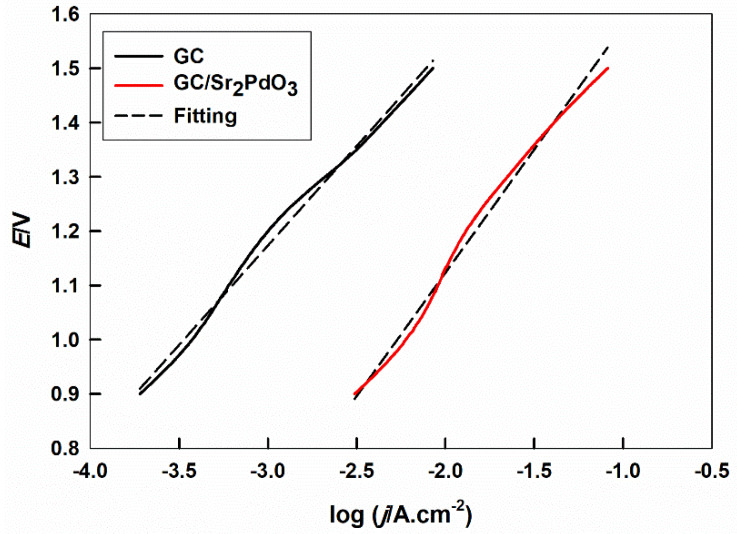
Potentiodynamic polarization of the unmodified and modified GC electrodes with Sr_2_PdO_3_ in a 0.1 M HClO_4_ solution.

The electrocatalytic behavior of Sr_2_PdO_3_, prepared by the citrate combustion approach for the OER in a 0.1 M HClO_4_ solution, is appraised by anodic PP. Figure 2 reveals PP curves for the bare GC and GC electrode, modified with the Sr_2_PdO_3_ perovskite, in the potential range of 0.9 to 1.5 V vs. Ag/AgCl. It can be detected that the current density, due to the OER, is increased by about 50 times in the presence of the Sr_2_PdO_3_ perovskite at a definite potential value of 1.0 V vs. Ag/AgCl. Giordano et al. reported the use of HfN as an efficient catalyst for OER, which gave a current density of about 5 mA cm^−2^ at 1.5 V [11]. Meanwhile, in our case the value of the current density, at 1.5 V, is 8.2 mA cm^−2^. This can be attributed to the catalytic activity of the Sr_2_PdO_3_ perovskite for the OER, resultant from the highly stabilized nano-Pd sites within the perovskite matrix [32,33,34,35], which utilizes its application as an efficient electrocatalyst for water oxidation.

According to the Tafel equation, Equation (1) [42]:

(1) $$ɳ=a+b\log\frac{I}{I_{o}}$$

where ɳ is the overpotential (volt), *I* is the current density (A cm^−2^), *b* is Tafel slope (volt), and *a* is the intercept which is related to the exchange current density, *I_o_*, by the Equations (2) and (3):

(2) $$a=-b\log I_{o}$$

(3) $$b=-\frac{2.3\;RT}{\propto F}$$

where *F* is Faraday constant (96,485 C mol^−1^), *R* is the universal gas constant (8.314 J.mol^−1^ K^−1^), *T* is the temperature in Kelvin, and *α* is the transfer coefficient.

The calculated values of the Tafel slope from Figure 2 are 366.3 and 454.1 mV for the bare GC and GC electrode modified with Sr_2_PdO_3_, respectively. The value of the Tafel slope usually ranges from 30 to 120 mV and can help to determine whether the slowest step of the reaction is from the first or second electron transfer or the recombination step. In our case, the higher Tafel slope values are observed, indicating additional contributions related to the oxide-surface processes, a potential drop in the anode charge layer, or a blockage of the electrode surface by the bubble accumulations [43,44,45,46]. The high Tafel slope values, 200 to 500 mV, were previously reported in literature for the same mentioned reasons [20]. The calculated values of the logarithm of the exchange current density, log *I_o_*, which is directly proportional to the rate of the reaction at equilibrium, are −6.2 and −4.4, (*I_o_* = 0.6 and 39.8 µA.cm^−2^) for the bare GC and GC electrode modified with Sr_2_PdO_3_, respectively. In other words, the reaction rate at equilibrium is highly increased upon the perovskite modification.

### 2.3. Determination of the Reaction Order

**Figure 3:**
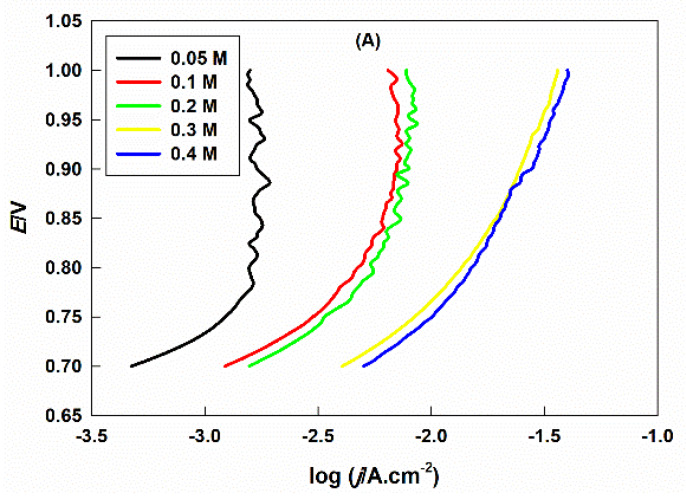

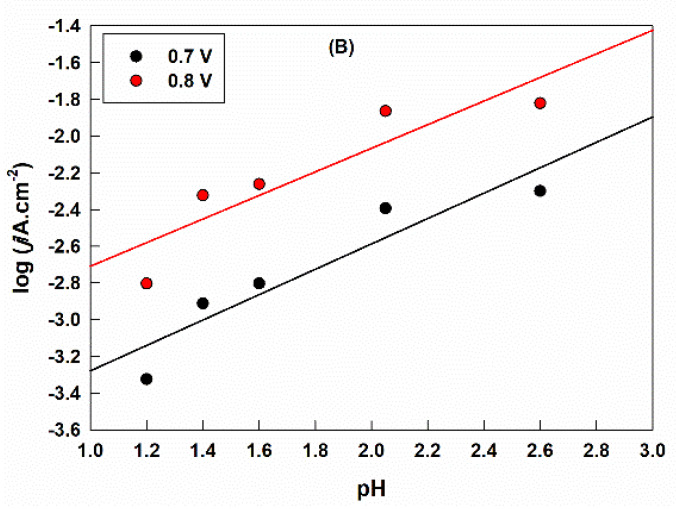
The (**A**) potentiodynamic polarization of GC/Sr_2_PdO_3_ in different concentrations of the HClO_4_ solutions and (**B**) the dependence of the logarithm of the current density at a definite potential on the pH of the solution.

Figure 3A indicates PP curves for the GC electrode modified with Sr_2_PdO_3_ in different concentrations of aqueous HClO_4_ (0.05 to 0.4 M) at a constant ionic strength by using Na_2_SO_4_. This can demonstrate that the rate of the OER is increased with increasing the concentration of the HClO_4_ solution. The reaction order can be determined from the slope of the logarithm of the current density at a certain potential value, at which a considerable OER is observed, against the pH value of the solution, Figure 3B. The reaction order is 0.81, which is close to unity [20]. This suggests that no further hydroxide ions are adsorbed from the bulk electrolyte before or during the rate-determining step [46,47,48].

### 2.4. Temperature Effect and Activation Energy Determination

**Figure 4:**
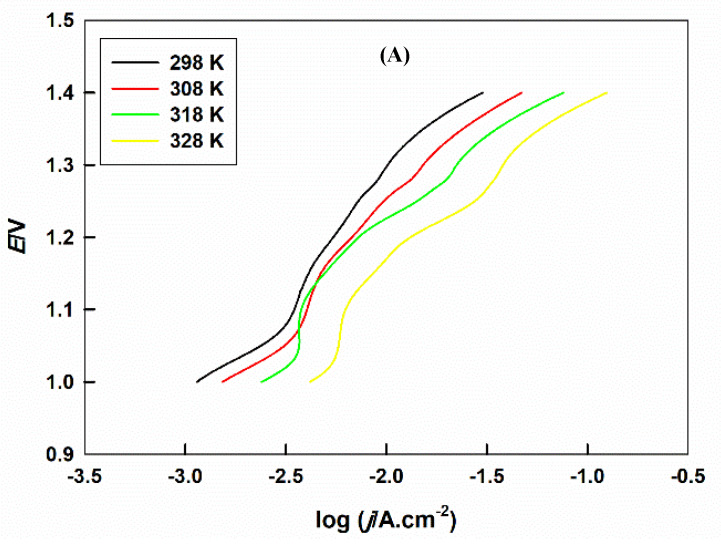

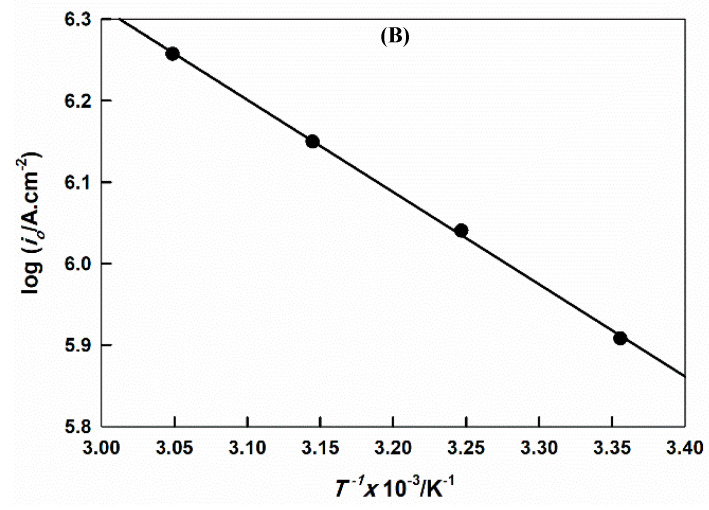
(**A**) The potentiodynamic polarization of GC/Sr_2_PdO_3_ at different temperatures in 0.1 M HClO_4_ solution, and (**B**) the corresponding Arrhenius plot.

The temperature effect on the rate of the OER at Sr_2_PdO_3_ in a 0.1 M HClO_4_ aqueous solution is performed by recording the PP curves at different temperatures (*T*), from 298 to 328 K, Figure 4A. The reaction rate is favored with the temperature rise. The value of the activation energy (*E*_a_) can be determined from the Arrhenius plot, Figure 4B, which equals the slope of log *I_o_* vs. $\frac{1}{T}$ plot, multiplied by −2.3 × *R*. The calculated value of *E*_a_ for the OER at Sr_2_PdO_3_ is 21.6 kJ mol^−1^, which can be considered a decent value in evaluation with those reported for the transition metal based perovskites, LaBO_3_ (B = Fe, Ni, Mn, Co, or Cr) tested in the same electrolyte, *E*_a_ values from 11.3 to 552.9 kJ mol^−1^ [20,21], or those of other OER perovskite catalysts in an alkaline medium, *E*_a_ values from 45.1 to 89.7 kJ mol^−1^ [49,50,51]. This reflects the high electrocatalytic activity for Sr_2_PdO_3_ toward the OER. This can be explained based on the matrix effect of the stable nano-perovskite crystal structure, in which nano-Pd sites are highly distributed and stabilized within the matrix [51,52,53,54].

### 2.5. Stability

**Figure 5:**
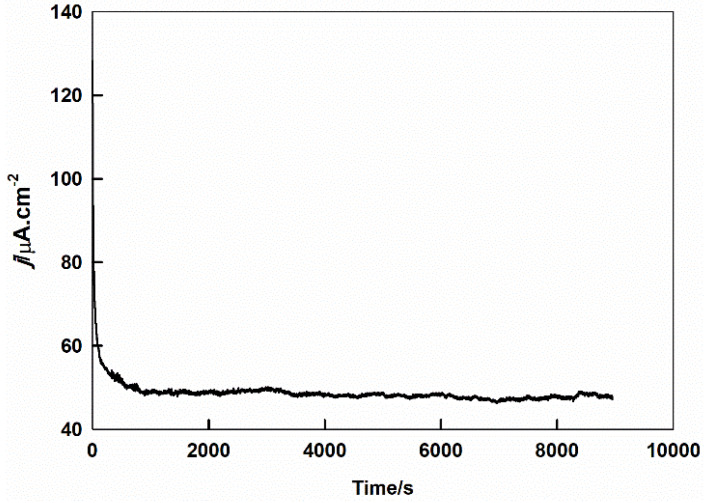
The current-time response of the potentiostatic experiment of GC/Sr_2_PdO_3_ in 0.1 M HClO_4_ subjected to a potential of 0.5 V for 150 min.

The stability of the proposed Sr_2_PdO_3_ catalyst for the OER in a 0.1 M HClO_4_ solution is examined by performing the reaction at a constant potential of 0.5 V and monitoring the current with the operation time. Figure 5 displays the current-time response of the GC electrode casted with Sr_2_PdO_3_ in a 0.1 M HClO_4_ aqueous solution by being subjected to a constant potential of 0.5 V for 150 min. A sharp current decrease is discerned during the first minutes, which arises from the decrease in the capacitive property. Subsequently, the current due to the OER is almost constant with the *%* decrease in the current at 2.5*%* after two and a half hours of the catalyst operation. This result reflects the high catalyst stability and excludes any probability of catalyst poisoning. Furthermore, the Sr_2_PdO_3_ perovskite presents an enhanced catalytic performance under potentiostatic conditions, as indicated by the increased current value 47.6 µA cm^−2^ in assessment with the potentiodynamic experiment, 31.6 µA cm^−2^. This can be explicated on the basis that the catalyst undergoes “self-activation” under potentiostatic conditions [53].

### 2.6. Electrochemical Impedance Spectroscopy (EIS)

The mechanism by which the OER occurs at the Sr_2_PdO_3_ surface can be analyzed by performing the EIS measurements, according to the classical Equation (4); the real part of the impedance, *Z’*, can be expressed as a function of the frequency *ω*:

(4) $$Z^{\prime }=R_{s}+R_{P}+R_{CT}+\sigma_{W}\;\surd \omega$$

where *R_s_*, *R_p_*, and *R_CT_* are solution, polarization, and charge-transfer resistances, respectively. *σ_W_* is the Warburg impedance.

**Figure 6:**
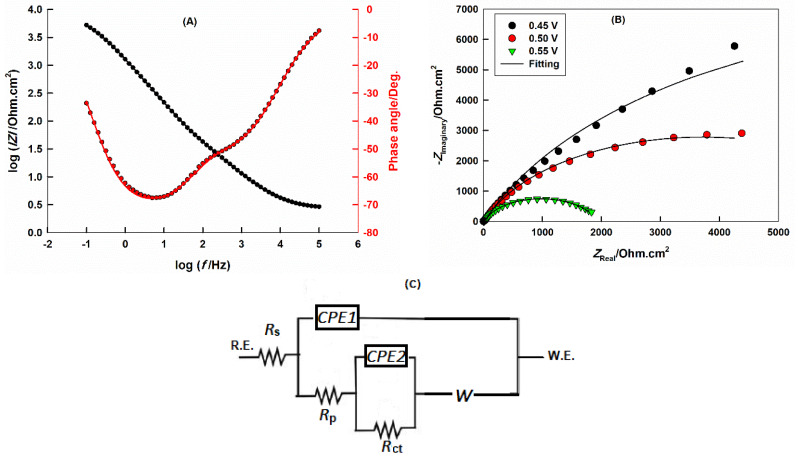
The (**A**) Bode and (**B**) Nyquist plots GC/Sr_2_PdO_3_ in 0.1 M HClO_4_ at 0.50 V and various overpotentials, respectively. Symbols are experimental and solid lines are modeled data. (**C**) is the electrical equivalent circuit used for fitting.

Figure 6 portrays the EIS spectra, in terms of the (A) Bode and (B) Nyquist plots, of the GC electrode casted with Sr_2_PdO_3_ in a 0.1 M HClO_4_ aqueous solution. The selected overpotential is 0.50 V in the case of the Bode plot and different values for the Nyquist plots: 0.45, 0.50, and 0.55 V. The EIS data reveals the presence of two time constants, as indicated by the two semicircles appearing in the Nyquist plots. The first semicircle, at high frequency regions, is related to the polarization and the double layer capacitance at the perovskite/electrolyte interfaces, *R*_p_ and CPE1, while the second semicircle, appearing at intermediate and low frequencies, is related to the charge-transfer and adsorption–desorption processes at the perovskite, *R*_CT_ and CPE2 [54,55,56,57]. The electrical equivalent circuit, illustrated in Figure 6C, is utilized for data fitting and exhibits a good agreement between experimental (circles) and fitted (lines) data. Similar circuits are employed successively for the study of the OER at metals and metal oxides surfaces [58,59]. The electrocatalytic activity of Sr_2_PdO_3_ for the OER was explored through the impact of the applied overpotential on the Nyquist plot, Figure 6B. The fitted parameters are presented in Table 1. It can be noted that with increasing the value of the applied overpotential, the value of *R*_CT_ is decreased, reflecting the high catalytic performance of Sr_2_PdO_3_ for the OER. It is worth mention that by increasing the applied overpotential, the diffusion within the perovskite matrix decreases quickly.

## 3. Materials and Method

### 3.1. Chemicals

Strontium nitrate (99%), palladium (II) chloride (99%), ammonium hydroxide (28–30% NH_3_ basis), perchloric acid (ACROS, 70%), nitric acid (70%), citric acid Anhydrous (99.5%), N,N-dimethyl formamide (DMF) (99%), and deionized water. All chemicals were purchased from the Sigma Aldrich Company.

### 3.2. Synthesis of Sr_2_PdO_3_ by the Citrate-Nitrate Combustion Method

The preparation through the sol-gel procedure has been reported in detail in a previous work [32,60,61,62,63], via mixing Sr(II) and Pd(II) ions in the same molar ratio. Subsequently, citric acid was added to the homogeneous metal ions solution, pH = 2. The solution was kept stirred while heating until complete vaporization, and then it was ignited. The resultant powder was calcined at 750 °C for 3 h to obtain a crystalline perovskite phase.

### 3.3. Electrochemical Cell and Measurements

The electrochemical measurements were performed in a one-compartment, three-electrode cell in which the working electrode was a glassy carbon (GC) electrode (area = 0.071 cm^2^), the auxiliary electrode was a platinum coil, and a saturated Ag/AgCl (3.5 M) was used as a reference electrode. The modified electrode, GC/Sr_2_PdO_3_, was prepared by casting 25 μL of the perovskite suspension in DMF, which was prepared by homogenously mixing 10 mg of perovskite in 1 mL of DMF.

The anodic PP measurements were done in a 0.1 M HClO_4_ aqueous solution by first operating an open circuit potential experiment for 10 min until a steady state was reached. This was followed by the conditioning of the working electrode under two-step potentiostatic conditions at 0.4 V for 300 s and at 0.5 V for 600 s, respectively. Finally, the appearance of the PP measure occurred from 0.5 to 1.5 V at a scan rate of 5 mV s^−1^. All the electrochemical parameters were calculated based on the ohmic drop correction.

The EIS was recorded at different applied overpotentials at a frequency range from 100,000 to 0.1 Hz with an amplitude of 5 mV. All the electrochemical measurements were performed employing a Gamry 1000 potentiostat.

### 3.4. Sample Characterizations

The X-ray diffractogram was acquired by XRD, Shimadzu, XRD-7000, Japan, at 40 kV and 30 mA, utilizing the CuK_α_ incident beam. The transmission electron microscope (TEM) image is taken by the TEM, JEOL JEM 1400, Japan.

## 4. Conclusions

Sr_2_PdO_3_, synthesized through the citrate-combustion methodology, was effectually employed as an electrocatalyst for the OER. The current density was amplified by about 50 times by casting the Sr_2_PdO_3_ perovskite on the GC substrate and values of *R*_CT_, calculated from the Nyquist plots fitting, and was decreased with the upsurge in the applied overpotentials, reflecting a powerful catalytic performance of Sr_2_PdO_3_ for the OER in 0.1 M HClO_4_ solution. This funding is substantiated by the low values of the calculated activation energy 21.6 kJ mol^−1^. Sr_2_PdO_3_ exhibited a high operation stability with the self-activation property, which promotes its use in water splitting fuel cells.

## Figures and Tables

**Table 1:** Fitting parameters obtained by using the electrical equivalent circuit, shown in Figure 6C, for Nyquist plots of GC/Sr_2_PdO_3_ in 0.1 M HClO_4_ solution, at different applied overpotentials.

Applied Overpotential (V)	*R*_s_ (Ω cm^2^)	CPE1 (µF cm^−2^)	*n*	*R*_P_ (Ω.cm^2^)	CPE2 (µF cm^−2^)	*m*	*R*_CT_ (kΩ.cm^2^)	*W* (mΩ s^−0.5^)
0.45	2.73	456.3	0.68	13.45	172.3	0.80	17.53	23.97
0.50	2.63	524.3	0.66	14.69	167.0	0.82	7.14	9.33
0.55	2.61	572.5	0.62	24.75	148.6	0.85	1.90	-
